# Polychlorinated Biphenyl Transformation, Peroxidase and Oxidase Activities of Fungi and Bacteria Isolated from a Historically Contaminated Site

**DOI:** 10.3390/microorganisms11081887

**Published:** 2023-07-26

**Authors:** Flavien Maucourt, Bastien Doumèche, David Chapulliot, Laurent Vallon, Sylvie Nazaret, Laurence Fraissinet-Tachet

**Affiliations:** 1 Université de Lyon, Universite Claude Bernard Lyon 1, CNRS, INRAE, VetAgro Sup, UMR Ecologie Microbienne, F-69622 Villeurbanne, France; 2 ENVISOL, 2-4 Rue Hector Berlioz, F-38110 La Tour du Pin, France; 3 Université de Lyon, Universite Claude Bernard Lyon 1, CNRS 5246 ICBMS, F-69622 Villeurbanne, France

**Keywords:** PCB, bioremediation, oxidase, peroxidase, microbes

## Abstract

Causing major health and ecological disturbances, polychlorinated biphenyls (PCBs) are persistent organic pollutants still recovered all over the world. Microbial PCB biotransformation is a promising technique for depollution, but the involved molecular mechanisms remain misunderstood. Ligninolytic enzymes are suspected to be involved in many PCB transformations, but their assessments remain scarce. To further inventory the capabilities of microbes to transform PCBs through their ligninolytic enzymes, we investigated the role of oxidase and peroxidase among a set of microorganisms isolated from a historically PCB-contaminated site. Among 29 isolated fungi and 17 bacteria, this work reports for the first time the PCB-transforming capabilities from fungi affiliated to *Didymella*, *Dothiora*, *Ilyonectria*, *Naganishia*, *Rhodoturula*, *Solicoccozyma*, *Thelebolus* and *Truncatella* genera and bacteria affiliated to *Peribacillus frigotolerans*, *Peribacillus muralis*, *Bacillus mycoides*, *Bacillus cereus*, *Bacillus toyonensis*, *Pseudarthrobacter* sp., *Pseudomonas chlororaphis*, *Erwinia aphidicola* and *Chryseobacterium defluvii*. In the same way, this is the first report of fungal isolates affiliated to the *Dothiora maculans* specie and *Cladosporium* genus that displayed oxidase (putatively laccase) and peroxidase activity, respectively, enhanced in the presence of PCBs (more than 4-fold and 20-fold, respectively, compared to controls). Based on these results, the observed activities are suspected to be involved in PCB transformation.

## 1. Introduction

Polychlorinated biphenyls (PCBs) are synthetic compounds manufactured by humans and used from 1929 to their prohibition at the end of 1980s. Mainly found in electrical transformer devices, paints, plasticizers or flame retardants [1], they were classified as persistent organic pollutants according to the Stockholm Convention from 2001. Breivik et al. (2002) have estimated that approximately 13 million tons of PCBs were synthetized worldwide, with 20% to 35% released in ecosystems [1,2]. Recognized as endocrine disruptors and carcinogenic substances [3,4], they represent ecological and health concerns. Due to their chemical characteristics, they are stable and persistent molecules that accumulate in ecosystems and the food web through their bioaccumulation and biomagnification, making their degradation a challenge [5,6].

Composed of biphenyl and chlorine atoms that differ in number (up to 10) and position on the phenyl rings, 209 theoretical chlorinated forms exist [6]. Currently, many physical and chemical methods have been developed to depollute PCB-contaminated matrices such as burning or use of solvents, but they are long, expensive and generate some toxic by-products. Therefore, bioremediation was simultaneously investigated [7]. This last technique emerged as a low-cost and theoretically healthier alternative and revealed the PCB-transforming capabilities of several microbes such as filamentous fungi and bacteria [8,9]. Because of their hyphal structure, also named “fungal highway”, filamentous fungi are suggested to be good candidates to easily penetrate polluted matrices in order to access poorly bio-accessible pollutants and also act as a dispersion vector for bacteria [10]. These PCB-transforming fungi are mainly ligninolytic *Ascomycota* and *Basidiomycota* from the white-rot functional guild. Among these white-rot fungi, the most well-known degrading species belong to *Bjerkandera adusta*, *Irpex lacteus*, *Pycnoporus cinnabarinus*, *Phanerochaete magnoliae* [11], *Phanerochaete chrysosporium* [12], *Lentinus edodes* [13], *Trametes versicolor* [14] and the most documented and efficient PCB transformers *Pleurotus ostreatus* [11,15]. Concerning the bacteria, only two bacterial metabolic pathways are described for PCB transformation. The first one occurs in anaerobic condition. Reductive dehalogenases (EC 1.97.1.8) are the key enzymes of this dechlorinated process and encoded by the *rdh* genes [16]. The prominent PCB-transforming anaerobic bacteria belong to the genera *Dehalogenimonas* and *Dehalococcoides* [8]. The second pathway named *bph* pathway (bph = biphenyl), confers the ability to use biphenyls as carbon sources [17] through the action of the biphenyl dioxygenase, a multi-component enzyme. However, the *bph* pathway may generate toxic dead-end products such as chlorobenzoic acid (CBA) [17]. In vitro studies showed its accumulation in culture media and the inability of many bacteria to grow with this subproduct [18]. The most frequently bacterial aerobic genera reported to transform PCBs are *Burkholderia*, *Pseudomonas*, *Comamonas*, *Sphingomonas*, *Cupriavidus*, *Rhodococcus*, *Acidovorax*, *Acinetobacter*, *Arthrobacter*, *Bacillus* and *Corynebacterium* [8].

Among the studies focusing on new PCB-transforming pathways, ligninolytic enzymes seem to be promising. These enzymes are represented by two groups named laccases and peroxidases and are produced by organisms including fungi and bacteria [19]. Whether produced by bacteria or fungi, they are suspected to be involved in PCB transformation. In addition to their metabolic versatility to transform many molecules structurally close to PCBs, such as chlorophenol, tetraguaïacol, methoxychlor [20,21,22,23,24,25], purified fungal laccases from *Coprinus comatus* [26], *Pycnoporus cinnabarinus*, *Myceliophthora thermophila* [27], *Cladosporium* sp. [28], *Trametes versicolor* and *Pleurotus ostreatus* [29,30], have been reported so far to transform PCBs *in vitro*. Fungal peroxidases from *Acremonium sclerotigenum* have also been observed to be more active in the presence of PCBs [31], and plant peroxidase displayed PCB-transforming capabilities [32]. Because data about this field remain scarce, more studies are needed to fully reveal the PCB-transforming capabilities of the ligninolytic enzymes.

To answer the question of the potential ability of fungal and bacterial species to be involved in PCB transformation through their laccase and peroxidase activities, we screened a collection of soil heterotrophic fungi and bacteria isolated from a long-term PCB-contaminated site. We analyzed (i) their ability to transform PCBs in individual isolate culture media, (ii) the presence of laccase and peroxidase encoding genes in their genomes versus *bph* genes for bacterial isolates and (iii) their capacity to produce and secrete active oxidases and peroxidases in culture media.

## 2. Materials and Methods

### 2.1. Microbial Isolation

The microorganisms used in this study were isolated from a PCB-contaminated brownfield (a former paint factory) where iPCB (see below) concentration reached 31 mg.kg^−1^ of dry soil. This brownfield is located at Pont-de-Claix, Isère, France (45,136578° N 5,697205° E) and managed by a collaborative society of collective interest, the SCIC CRISALID. Ten soil cores were randomly sampled on a sampling grid and separated by at least 4 m in the brownfield. Briefly, 375 g of sieved soil was collected for each soil sample, and a homogenized aliquot of 2 g from each sample was suspended in 5 mL of NaCl (1.2 M, 70% *w*/*v*) solution, diluted until 10^−3^ dilution, spread on solid Dichloran Rose Bengale Chloramphenicol (DRBC) medium or solid CYM media and incubated at 24 °C for fungi and on solid LB medium at 30 °C for bacteria. One liter of DRBC medium was made of polypeptone (5 g), glucose (10 g), monopotassium phosphate (1 g), MgSO_4_ (0.5 g), dichloran (0.2 g), rose bengale (0.25 g), chloramphenicol (0.5 g), chlortetracycline (0.5 g), ZnSO_4_ (0.1 g), CuSO_4_ (0.05 g), tergitol (1 mL), agar (12.4 g). Solid CYM media contained the following for 1 L: maltose (10 g), glucose (20 g), yeast extract (2 g), KH_2_PO_4_ (4.6 g), MgSO_4_ (0.5 g), agar (15 g). Furthermore, 146 fungi and 83 bacterial colonies were then isolated, all based on their morphotype differences to optimize taxonomic diversity.

### 2.2. PCR Amplification, Gene Sequencing and Taxonomic Affiliation

Fungal DNA extractions were conducted for each isolate as described by Liu et al. [33]. The PCRs were carried out according to the conventional protocols using a Biorad C1000 thermal cycler (Biorad, Hercules, CA, USA), using 4 µL of HOT FIREPol^®^ Blend Master Mix Ready to Load (5X) (Solis Biodyne, Tartu, Estonia), 0.8 μM of each primer and 20 ng of fungal genomic DNA or small amount of a bacterial colony picked up using a sterile toothpick, in a final volume of 25 μL. ITS and 16S rDNA amplicons, obtained with the ITS1F (5′-CTTGGTVATTTAGAGGAAGTAA-3′) and ITS4 (5′-TCCTCCGCTTATTGATATGC-3′) primer pair and the pA (5′-AGAGTTTGATCCTCGCTCAG-3′) and pH (5′-AAGGAGGTGATCCAGCCGCA-3′) primer pair, respectively, were sequenced (Microsynth France, Vaulx-en-Velin, France https://www.microsynth.com/standard-services.html, accessed on 19 April 2023). For taxonomic affiliation, fungal isolates were first identified according to general morphotype principles of fungal classification. Then, ITS sequences were compared against tree databases: the rRNA/ITS databases with fungi (taxid:4751) as organism option using Blastn+ [34]; the UNITE and the INSD databases using massBLASTer (BLAST+ 2.13.0) in PlutoF [35]. For bacteria, the 16S rDNA sequences were compared to the Refseq genome database using Blastn+ [34]. The 16S rDNA sequences were further aligned with reference sequences and phylogenetic trees using the Maximum Likelihood method, and a Kimura 2-parameter model with 1000 replicates was constructed using Seaview [36]. Amplicons of laccase-encoding genes were obtained using the degenerate primer pairs Cu1BF/Cu2R targeting laccase-encoding genes from *Basidiomycota* and Cu1AF/Cu2R targeting *Ascomycota* ones [37]. Fungal dye-peroxidases and *Basidiomycota* class II peroxidase genes were amplified using DYP360F/DYP485R [38] and AA2-F and AA2-R [39] primer pairs, respectively. The F_DYPR and R_DYPR primers from Tian et al. 2016 were used to target bacterial Dye-Peroxidase genes [40]. The bacterial *bphA* gene was amplified using the 4 different pairs of primers from Correa et al. 2010 [41]. All positive amplicons were cloned using TOPO ^®^ TA Cloning^®^ Kit for Sequencing (Invitrogen, Waltham, MA, USA), and ten clones per amplicon were analyzed after sequencing by Microsynth France.

### 2.3. PCB Transformation Analysis

Each fungal isolate was grown on Petri dishes with CYM media. In triplicate, 10 calibrated fresh mycelial inocula (diameter 5 mm) were added to a liquid minimal media [42,43]. After incubation for 2 days at 24 °C on a rotary shaker (120 rpm), Aroclor 1254 (25 mg·L^−1^ dissolved in methanol (Sigma-Aldrich^®^, Saint Quentin Fallavier, France) was added, and each culture was incubated for 3 additional days under the same conditions. After incubation, cultures were frozen at −80 °C. Each bacterial isolate was cultivated in quadruplicate in liquid LB medium until mid-exponential growth time and washed 3-times with NaCl 0.9% (1.2 M, 70% *w*/*v*) solution. Then, 50% of washed cells were transferred in glass tube containing 5 mL of a media (Yeast extract 10 g·L^−1^, NH_4_Cl 2 g·L^−1^, NaCl 1 g·L^−1^, KH_2_PO_4_ 6 g·L^−1^, Na_2_HPO_4_ 25.6 g·L^−1^ and Aroclor 1254 25 mg·L^−1^ (Sigma-Aldrich, dissolved in methanol)), incubated during 48 h at 30 °C on a rotary shaker (120 rpm). After incubation, cultures were frozen at −80 °C. Indicator PCB (iPCB) assays of the whole culture (including extracellular medium and microbial cells) were performed using gas chromatography coupled with mass spectrometry (GC-MS) (AGROLAB FRANCE SARL, Dijon, France). Positive controls without fungal or bacterial cells and with Aroclor 1254 were also assayed in triplicate. iPCBs correspond to PCB congeners 28, 52, 101, 118, 138, 153 and 180 found to be the most abundant PCB congeners in environmental and biological matrices and the most common and easily determined congeners [44]. The percent of iPCB transformation efficiency was calculated for each isolate by subtracting the average iPCB quantity of each isolate to the positive control. The results were then transformed in percentages by considering control as 100%. Comparison of iPCB amount between controls and biotransformation assays was conducted using a Pairwise Wilcoxon test using the R software (https://www.r-project.org/, accessed on 18 November 2023).

### 2.4. Oxidase and Peroxidase Activity Assay

Each fungal isolate was cultivated as described above except that minimum media was supplemented with Aroclor 1254 1 mg·L^−1^ (dissolved in 5 µL methanol), methanol (5 µL) or no supplementation for control conditions. Each bacterial isolate was cultivated in a media containing Yeast Carbon Base—NutriSelect^®^ 11.7 g·L^−1^, (NH_4_)_2_SO_4_ 3.5 g·L^−1^, CH_3_COCOOH 10 g·L^−1^ supplemented with Aroclor 1254 1 mg·L^−1^ (dissolved in 5 µL methanol), methanol (5 µL) or no supplementation for both control conditions. After a total of 7 days of incubation, cells were centrifuged 10 min at 4000 g. For oxidase activity assay, 50 µL of supernatant was transferred in 96-well plate preloaded with 200 µL of phosphate citrate buffer pH 4 containing ABTS (2,2’-azino-bis(3-ethylbenzothiazoline-6-sulfonic acid)) (5.8 mM) or Guaiacol (53 mM) as substrates (Sigma-Aldrich^®^). The same protocol was used for peroxidase activity except that H_2_O_2_ (100 µM) was added to each well. Oxidase and peroxidase activities were quantitatively determined by measuring absorption change of each substrate for 4 h using TECAN infinite 200^®^ PRO microtiter plate reader (Tecan, Salzburg, Austria) at 740 nm and 470 nm for ABTS and Guaiacol, respectively. Molar extinction coefficients of ABTS and Guaiacol (ɛ_M_) were obtained by measuring their full oxidation in the concentration range from 10 µM to 1000 µM at the previously mentioned wavelength. The estimation of total excreted proteins was measured with Coomassie (Bradford) Protein Assay Kit (Thermo Scientific, Illkirch, France). Specific activities are expressed in μmol of substrate oxidized per minute and per mg of total excreted proteins (µmol·min^−1^·µg^−1^). A pairwise Wilcoxon test in R software was conducted to compare oxidase activities measured in the presence of PCBs compared to controls (with or without methanol).

## 3. Results

First, 146 fungi and 83 bacteria were isolated from a PCB-contaminated brownfield, taxonomically characterized and assayed for their PCB-transformation capacity. Among them, the 29 fungal isolates listed in Table 1 and the 17 bacterial isolates listed in Table 2 were selected with respect to their taxonomic diversity and for their highest PCB-transformation efficiency, i.e., presenting more than 30% or 10% of iPCB depletion in 3 or 2 days for fungi or bacteria, respectively.

Regarding the fungal taxonomic affiliations, most of the isolates (24 exactly) are affiliated to *Ascomycota* phylum. They belong to *Alternaria* sp. (CRIS_F20 and F22), *Aspergillus* sp. (CRIS_F8, F11, F12 and F23), *Cladosporium* sp. (isolates CRIS_F24, F25, F26, F27 and F29), *Didymella* sp. (CRIS_F16), *Dothiora maculans* (CRIS_F2, F9 and F13), *Fusarium* sp. (CRIS_F5, F6), *F. oxysporum* (CRIS_F10), *Ilyonectria* sp. (CRIS_F28), *Penicillium* sp. (CRIS_F3), *Thelebolus* sp. (CRIS_F14), *Trichoderma* sp. (CRIS_F7), *T. harzanium* (CRIS_F18) and *Truncatella angustata* (CRIS_F19). The 5 remaining fungi, all isolated in their yeast state, are affiliated to *Basidiomycota* phylum and belong to *Naganishia* sp. (isolates CRIS_F4, and F17), *Rhodoturula mucilaginosa* (CRIS_F1), *Solicoccozyma* sp. (CRIS_F15) and *S. aeria* (CRIS_F21). 

**Figure 1:**
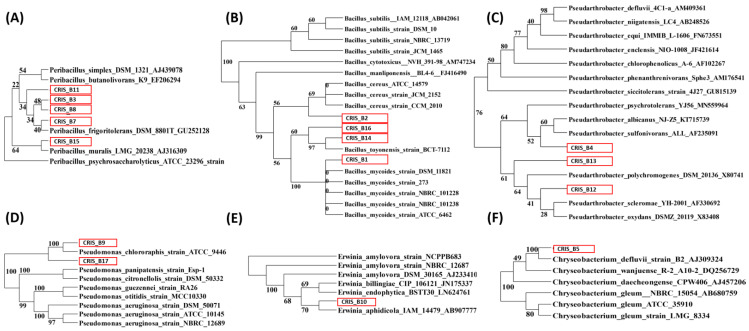
Phylogenetic trees by Maximum Likelihood method and Kimura 2-parameter based on 16S rRNA gene sequences of the isolates from this study surrounded by red boxes and reference sequences from genera (**A**) *Peribacillus,* (**B**) *Bacillus,* (**C**) *Pseudarthrobacter,* (**D**) *Pseudomonas,* (**E**) *Erwinia,* (**F**) *Chryseobacterium*. Bootstrap values shown at branch nodes are generated from 1000 replicates and expressed as percentage.

Regarding the blast bacterial species affiliation (Table 2) and the bacterial phylogenetic trees (Figure 1), the bacterial isolates are phylogenetically close to *Arthrobacter* sp. (CRIS_B6), *Bacillus cereus* (CRIS_B2—Figure 1B), *B*. *mycoides* (CRIS_B1—Figure 1B), *B. toyonensis* (CRIS_B14 and B16—Figure 1B), *Chryseobacterium defluvii* (CRIS_B5—Figure 1F), *Erwinia aphidicola* (CRIS_B10—Figure 1E), *Peribacillus frigoritolerans* (CRIS_B7, B8 and B10—Figure 1A), *Peribacillus muralis* (CRIS_B15—Figure 1A), *Pseudarthrobacter* sp. (CRIS_B4, B12 and B13—Figure 1C), *Pseudomonas chlororaphis* (CRIS_B9—Figure 1D) and *Pseudomonas* sp. (CRIS_B17—Figure 1D). Their iPCB transformation efficiencies vary from 31.6% (±10.2%) to 96.2% (±2.84%) for fungal isolates (Table 1) and from 17.4% (±3.49) to a maximum of 58% (±8.65) for bacterial isolates (Table 2) supported by a pairwise Wilcoxon test indicated significant differences (*p*-value < 0.001) between controls and biotransformation assays. It is worth noting that isolates able to transform iPCBs with an efficiency of more than 90% mainly belong to *Cladosporium* genera. Concerning genomic DNA ligninolytic gene amplification confirmed by sequencing, laccase encoding genes were amplified in only 6 fungal isolates belonging to *Dothiora* sp. and *Dothiora maculans* (isolates CRIS_F2 and F13, respectively), *Fusarium* sp. (CRIS_F6), *Alternaria* sp. (CRIS_F20 and F22) and *Cladosporium* sp. (CRIS_F27). In the same way, dye-peroxidase encoding gene was observed for only one fungal isolate (CRIS_F12) belonging to *Aspergillus*. Concerning the bacterial isolates, no ligninolytic and *bph* enzyme-encoding gene was amplified.

**Figure 2:**
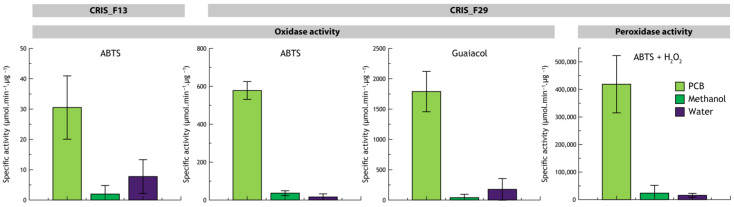
Oxidases and peroxidases specific activities of CRIS_F13 and CRIS_F29, using ABTS, guaiacol (oxidase activity) or ABTS and hydrogen peroxide (peroxidase activity) regarding the presence of PCB (in methanol), methanol or no additive during the culture.

Regarding oxidase activities, fungal isolates CRIS_F2, F8, F18, F20, F22, F24, F25, F26 and CRIS_F27, respectively affiliated to *Dothiora* sp., *Aspergillus* sp., *Trichoderma harzianum*, *Alternaria* sp., *Alternaria* sp. and *Cladosporium* sp. for the last three, showed average oxidase activities in any conditions for ABTS and/or Guaiacol varying from 15.21 to 255.92 µmol·min^−1^·µg^−1^. Average oxidase activities (ABTS as substrate) from fungal isolates CRIS_F29 and CRIS_F13, respectively affiliated to *Cladosporium* sp. and *Dothiora maculans*, were both enhanced in the presence of PCBs (578.01 and 30.50 µmol·min^−1^·µg^−1^, respectively) compared to controls (36.42 and 1.96 µmol·min^−1^·µg^−1^ in the presence of methanol only, respectively, and 16.19 and 7.73 µmol·min^−1^·µg^−1^ for the control without additive, respectively), supported by a *p*-value of 0.029 and 0.0065, respectively (Figure 2). Guaiacol oxidase activity was enhanced only from the fungal isolate CRIS_F29 in the presence of PCBs (1.7 × 10^2^ µmol·min^−1^·µg^−1^) compared to controls (39.48 and 623.8 µmol·min^−1^·µg^−1^ for control in the presence of methanol only and control without additives, respectively) supported by a *p*-value of 0.019 (Figure 2). Regarding peroxidase, ABTS peroxidase activity was enhanced only from the *Cladosporium* sp. isolate, CRIS_F29, in the presence of PCBs (4.1 × 10^5^ µmol·min^−1^·µg^−1^) compared to controls (2.3 × 10^4^ and 1.5 × 10^4^ µmol·min^−1^·µg^−1^ for control in the presence of methanol only and control without additives, respectively) supported by a *p*-value of 0.0055 (Figure 2). All other fungal isolates (up to 18) were without oxidase or peroxidase activity. Bacterial isolates showed neither oxidase nor peroxidase activity.

## 4. Discussion

While PCBs remain a major health and ecological issue, microbial PCB bioremediation processes are an attractive way to depollute contaminated matrices. However, molecular mechanisms performed by microbes to transform PCBs are still misunderstood. Among the new PCB-transforming pathway, ligninolytic enzymes that are produced by fungi and bacteria are good candidates to be responsible for PCB transformation. Indeed, several fungal purified laccases have been observed to transform some PCBs [19,27,28,45,46]. In this way, to further investigate the distribution of potential PCB-transforming laccases and peroxidases among fungal and bacterial taxa, the present work aimed to inventory microbial isolates involved in PCB transformation, taxonomically characterize them, amplify ligninolytic genes on their genomic DNA and measure their secreted oxidase and peroxidase activities in the presence and the absence of PCBs. In addition, the *bph* gene PCR amplification was performed on bacterial DNA in order to determine which bacterial isolate is able to transform PCB through the well-known *bph* pathway and those potentially using another biochemical pathway.

Among the 29 fungal isolates analyzed, this is the first report of PCB transformation ability for fungal isolates belonging to *Didymella*, *Dothiora*, *Ilyonectria*, *Naganishia*, *Rhodoturula*, *Solicoccozyma*, *Thelebolus* and *Truncatella* genera (Table 1). In the same way, among the 17 bacterial isolates, this is the first report of PCB-transforming capabilities from bacteria phylogenetically close to *Peribacillus frigotolerans*, *Peribacillus muralis*, *Bacillus mycoides*, *Bacillus cereus*, *Bacillus toyonensis*, *Pseudarthrobacter* sp., *Pseudomonas chlororaphis*, *Erwinia aphidicola* and *Chryseobacterium defluvii* (Table 2 and Figure 1). This is also the first report of PCB-enhanced peroxidase activities from a fungal isolate affiliated to *Cladosporium* sp. and PCB-enhanced oxidase activities from an isolate closely related to *Dothiora maculans* (more than 20 and 4-fold, respectively). Concerning fungal peroxidases and according to the literature, only one study reports enhanced peroxidase activity by PCBs from *Acremonium sclerotigenum* [31]. This supports the need of more investigation regarding peroxidase activity from fungi with PCB-transforming capabilities that might reveal a new PCB transforming pathway through peroxidases. On the contrary, laccases (that are oxidases) activities from *Pleurotus ostreatus*, *Fusarium solani*, *Thermothelomyces thermophila*, *Pleurotus sajor-caju* LBM 105, *Cladosporium* sp. and *Trametes versicolor* have been already observed to be enhanced by PCBs [28,31,43,47,48]. In this way, the enhanced oxidase activity in the presence of PCBs from fungal isolates CRIS_F13 phylogenetically close to *Dothiora maculans* and from CRIS_F29 affiliated to *Cladosporium* sp. suggests that this measured oxidase activity is likely due to the presence of laccases. This hypothesis is supported by the laccase gene amplification from the fungal isolate CRIS_F13. These PCB-enhanced peroxidase and oxidase activities also suggest that the PCB transformation observed for these two isolates might be due to laccases and peroxidases. The substrate proximity of laccase and peroxidase and purified laccases that displayed PCB-transforming abilities supports this hypothesis [26,27,28,29,30]. In addition, even if laccase activities of fungal isolates CRIS_F2, F8, F18, F20 F22, F24, F25, F26 and CRIS_F27 are not specifically enhanced by PCBs, they might be involved in observed PCB transformations. In any case, these hypotheses must be confirmed by testing purified laccases and peroxidases from those fungal isolates in PCB-transformation assays. 

Concerning the bacterial isolates, it is surprising that no *bph* gene amplification was observed among those affiliated to *Pseudomonas* sp. (CRIS_B17), *Pseudomonas chlororaphis* (CRIS_B9), *Peribacillus frigoritolerans* (CRIS_B3, B7, B8 and B11), *Bacillus cereus* (CRIS_B1), *B. mycoides* (CRIS_B2) and *B. toyonensis* (CRIS_B14 and B16). Indeed, there is a wide distribution of *bph* gene in *Pseudomonas* and *Bacillus* PCB-transforming bacteria [49,50,51], and the recent new *Peribacillus* genus was formerly *Bacillus* [52]. This suggests that primers used in this study might not be able to amplify all *bph* genes, or that still unknown PCB-transforming pathways are involved in the PCB-transformation realized by those isolates.

This last hypothesis can also be applied to the other fungal and bacterial isolates that transform PCB with the lack of oxidase and peroxidase activities or *bph* gene (for bacteria only). It is particularly true for the first report through this work of the PCB-transforming fungal isolates affiliated to *Didymella*, *Ilyonectria*, *Naganishia*, *Rhodoturula*, *Solicoccozyma*, *Thelebolus* and *Truncatella* genera and to the PCB-transforming bacterial isolates affiliated to *Chryseobacterium defluvii* and *Pseudarthrobacter* sp. with no *bph* genes reported so far for phylogenetically close strains. This highlights that further investigations about the PCB-transforming capabilities of these species are required to reveal new PCB-transforming pathways and to participate to better understand the PCB biotransformation process operated by the microorganisms.

## 5. Conclusions

For the first time, this study reports direct PCB transformation capabilities of fungal isolates belonging to *Didymella*, *Dothiora*, *Ilyonectria*, *Naganishia*, *Rhodoturula*, *Solicoccozyma*, *Thelebolus* and *Truncatella* genera and to bacterial isolates affiliated to *Peribacillus frigotolerans*, *Bacillus mycoides*, *B. cereus*, *B. toyonensis*, *Pseudarthrobacter* sp., *Pseudomonas chlororaphis*, *Chryseobacterium defluvii Erwinia aphidicola* and *Peribacillus muralis*. Concerning oxidase and peroxidase activities, only two fungal isolates showed PCB-enhanced activities with a first report of PCB-enhanced peroxidase activity from a fungal isolate affiliated to *Cladosporium* sp. and PCB-enhanced oxidase activity from another closely related to *Dothiora maculans* (more than 20 and 4-fold respectively). These activities suggest the involvement of laccases and peroxidases in the PCB-transforming action of those fungal isolates. The unobserved oxidase and peroxidase activities for 18 fungal isolates out of 29 and all the bacteria (added to no *bph* gene amplification for these last), suggest that still-unknown PCB-transformation pathways operate. In this way, further investigations focusing on PCB-transforming capabilities are required to reveal new PCB-transformation pathways, especially for fungal isolates affiliated to *Didymella*, *Ilyonectria*, *Naganishia*, *Rhodoturula*, *Solicoccozyma*, *Thelebolus* and *Truncatella* genera and to the PCB-transforming bacterial isolates affiliated to *Chryseobacterium defluvii* and *Pseudarthrobacter* sp. The next step of this work is to characterize at the species level the most efficient iPCB transformers and those showing an enhanced enzymatic activity in the presence of PCBs. For this purpose, PCB up-regulated peroxidases and laccases encoding genes from these isolates will be identified by RNA seq to further predict in silico how they could transform iPCBs by modeling Protein Ligand Docking on PCBs and up-regulated enzymes. Other enzyme encoding genes that could be identified as possible PCB transformers will undergo the same process. Then, the enzyme-PCB binding would have to be confirmed in vitro using purified enzymes from these isolates and by chemically characterizing the releasing PCB byproducts.

## Figures and Tables

**Table 1:** Identification name (ID), *phylum* and species blast taxonomic affiliation of the ITS region (with percent of identity), the database used for taxonomic affiliation and average degradation efficiency ± the standard deviation in percent for each assayed isolate.

ID	Phylum	Blast Result (% Identity)		Data Base	iPCB Degradation Efficiency (%)
CRIS_F1	*Basidiomycota*	*Rhodoturula mucilaginosa*	(100%)	INSD	31.63 ± 10.2
CRIS_F2	*Ascomycota*	*Dothiora* sp.	(97%)	NCBI	48.58 ± 16
CRIS_F3	*Ascomycota*	*Penicillium* sp.	(100%)	UNITE	56.49 ± 15.1
CRIS_F4	*Basidiomycota*	*Naganishia* sp.	(99.83%)	NCBI	62.71 ± 11.1
CRIS_F5	*Ascomycota*	*Fusarium* sp.	(100%)	INSD	64.06 ± 19
CRIS_F6	*Ascomycota*	*Fusarium* sp.	(100%)	INSD	64.4 ± 11.8
CRIS_F7	*Ascomycota*	*Trichoderma* sp.	(100%)	NCBI	65.36 ± 8.02
CRIS_F8	*Ascomycota*	*Aspergillus* sp.	(100%)	NCBI	66.44 ± 25.1
CRIS_F9	*Ascomycota*	*Dothiora maculans*	(100%)	INSD	68.36 ± 14.6
CRIS_F10	*Ascomycota*	*Fusarium oxysporum*	(100%)	INSD	72.54 ± 21.6
CRIS_F11	*Ascomycota*	*Aspergillus* sp.	(100%)	NCBI	72.76 ± 19.3
CRIS_F12	*Ascomycota*	*Aspergillus* sp.	(100%)	NCBI	76.61 ± 20
CRIS_F13	*Ascomycota*	*Dothiora maculans*	(100%)	INSD	77.01 ± 14.8
CRIS_F14	*Ascomycota*	*Thelebolus* sp.	(100%)	INSD	78.53 ± 9.29
CRIS_F15	*Basidiomycota*	*Solicoccozyma* sp.	(97.85%)	INSD	79.37 ± 7.4
CRIS_F16	*Ascomycota*	*Didymella* sp.	(99.81%)	INSD	82.82 ± 13.5
CRIS_F17	*Basidiomycota*	*Naganishia* sp.	(100%)	NCBI	83.61 ± 1.19
CRIS_F18	*Ascomycota*	*Trichoderma harzanium*	(100%)	INSD	85.72 ± 14.9
CRIS_F19	*Ascomycota*	*Truncatella angustata*	(100%)	NCBI	85.87 ± 14.2
CRIS_F20	*Ascomycota*	*Alternaria* sp.	(99.66%)	INSD	86.55 ± 14.8
CRIS_F21	*Basidiomycota*	*Solicoccozyma aeria*	(100%)	NCBI	88.13 ± 11.8
CRIS_F22	*Ascomycota*	*Alternaria* sp.	(100%)	NCBI	88.47 ± 5.11
CRIS_F23	*Ascomycota*	*Aspergillus* sp.	(100%)	NCBI	88.87 ± 5.24
CRIS_F24	*Ascomycota*	*Cladosporium* sp.	(100%)	NCBI	90.86 ± 7.88
CRIS_F25	*Ascomycota*	*Cladosporium* sp.	(100%)	NCBI	92.38 ± 6.49
CRIS_F26	*Ascomycota*	*Cladosporium* sp.	(100%)	NCBI	92.48 ± 1.92
CRIS_F27	*Ascomycota*	*Cladosporium* sp.	(100%)	NCBI	92.71 ± 1.88
CRIS_F28	*Ascomycota*	*Ilyonectria* sp.	(99.25%)	NCBI	94.6 ± 7.97
CRIS_F29	*Ascomycota*	*Cladosporium* sp.	(100%)	NCBI	96.29 ± 2.84

**Table 2:** Identification name (ID) and blast taxonomic affiliation of the 16S rRNA gene (with percentage of identity) for each assayed isolate with remaining iPCB concentration, transformation efficiency and *bphA* presence.

ID	Blast Result (% Identity)		iPCB µg·L^−1^ (Mean ± sd)	Transformation Efficiency (%)
Control	-		5197 ± 390	-
CRIS_B1	*Bacillus mycoides*	(99.80%)	4292 ± 181.41	17.4 ± 3.49
CRIS_B2	*Bacillus cereus*	(99.87%)	3935.83 ± 43.69	24.3 ± 0.84
CRIS_B3	*Peribacillus frigoritolerans*	(99.60%)	3602.5 ± 192.62	30.7 ± 3.71
CRIS_B4	*Pseudarthrobacter* sp.	(99.46%)	3595.35 ± 602.86	30.8 ± 11.6
CRIS_B5	*Chryseobacterium defluvii*	(100%)	3339.93 ± 828.61	35.7 ± 15.94
CRIS_B6	*Arthrobacter* sp.	(99.60%)	3227.03 ± 195.80	37.9 ± 3.77
CRIS_B7	*Peribacillus frigoritolerans*	(99.60%)	3194.7 ± 220.76	38.5 ± 4.25
CRIS_B8	*Peribacillus frigoritolerans*	(99.67%)	3103.25 ± 200.71	40.3 ± 3.86
CRIS_B9	*Pseudomonas chlororaphis*	(99.87%)	3085.86 ± 465.57	40.6 ± 8.96
CRIS_B10	*Erwinia aphidicola*	(99.47%)	3046.66 ± 790.34	41.4 ± 15.21
CRIS_B11	*Peribacillus frigoritolerans*	(99.67%)	3023.3 ± 388.43	41.8 ± 7.47
CRIS_B12	*Pseudarthrobacter* sp.	(99.73%)	2956.33 ± 575.33	43.1 ± 11.07
CRIS_B13	*Pseudarthrobacter* sp.	(99.60%)	2950.46 ± 481.88	43.2 ± 9.27
CRIS_B14	*Bacillus toyonensis*	(99.80%)	2925.33 ± 283.60	43.7 ± 5.46
CRIS_B15	*Peribacillus muralis*	(99.67%)	2887.5 ± 205.95	44.4 ± 3.96
CRIS_B16	*Bacillus toyonensis*	(99.60%)	2404.76 ± 512.85	53.7 ± 9.87
CRIS_B17	*Pseudomonas* sp.	(99.80%)	2180.16 ± 449.42	58 ± 8.65

## Data Availability

All data that support findings of this study have been deposited in National Center for Biotechnology Information (NCBI) under reference PRJNA924245 (https://www.ncbi.nlm.nih.gov/bioproject/PRJNA924245, accessed on 16 January 2023) for fungal isolates and under reference PRJNA924227 (https://www.ncbi.nlm.nih.gov/bioproject/PRJNA924227, accessed on 16 January 2023) for bacterial isolates.

## References

[1] Melymuk L., Blumenthal J., Sáňka O., Shu-Yin A., Singla V., Šebková K., Pullen Fedinick K., Diamond M.L. (2022). Persistent Problem: Global Challenges to Managing PCBs. Environ. Sci. Technol..

[2] Breivik K., Sweetman A., Pacyna J., Jones K. (2002). Towards a Global Historical Emission Inventory for Selected PCB Congeners—A Mass Balance Approach1. Global Production and Consumption. Sci. Total Environ..

[3] Faroon O.M., Samuel Keith L., Smith-Simon C., De Rosa C.T., World Health Organization (2003). Polychlorinated Biphenyls: Human Health Aspects.

[4] Pointing S. (2001). Feasibility of Bioremediation by White-Rot Fungi. Appl. Microbiol. Biotechnol..

[5] Fagervold S.K., May H.D., Sowers K.R. (2007). Microbial Reductive Dechlorination of Aroclor 1260 in Baltimore Harbor Sediment Microcosms Is Catalyzed by Three Phylotypes within the Phylum *Chloroflexi*. Appl. Environ. Microbiol..

[6] Maucourt F., Cébron A., Budzinski H., Le Menach K., Peluhet L., Czarnes S., Melayah D., Chapulliot D., Vallon L., Plassart G. (2023). Prokaryotic, Microeukaryotic, and Fungal Composition in a Long-Term Polychlorinated Biphenyl-Contaminated Brownfield. Microb. Ecol..

[7] Arbon R.E., Mincher B.J., Knighton W.B. (1994). .gamma.-Ray Destruction of Individual PCB Congeners in Neutral 2-Propanol. Environ. Sci. Technol..

[8] Khalid F., Hashmi M.Z., Jamil N., Qadir A., Ali M.I. (2021). Microbial and Enzymatic Degradation of PCBs from E-Waste-Contaminated Sites: A Review. Environ. Sci. Pollut. Res..

[9] Pieper D.H., Seeger M. (2008). Bacterial Metabolism of Polychlorinated Biphenyls. J. Mol. Microbiol. Biotechnol..

[10] Kohlmeier S., Smits T.H.M., Ford R.M., Keel C., Harms H., Wick L.Y. (2005). Taking the Fungal Highway: Mobilization of Pollutant-Degrading Bacteria by Fungi. Environ. Sci. Technol..

[11] Čvančarová M., Křesinová Z., Filipová A., Covino S., Cajthaml T. (2012). Biodegradation of PCBs by Ligninolytic Fungi and Characterization of the Degradation Products. Chemosphere.

[12] Kamei I., Kogura R., Kondo R. (2006). Metabolism of 4,4′-Dichlorobiphenyl by White-Rot Fungi *Phanerochaete chrysosporium* and *Phanerochaete* sp. MZ142. Appl. Microbiol. Biotechnol..

[13] Ruiz-aguilar G.M.L., Fern J.M., Rodrıguez-vazquez R., Poggi-Varaldo H. (2002). Degradation by White-Rot Fungi of High Concentrations of PCB Extracted from a Contaminated Soil. Adv. Environ. Res..

[14] Plačková M., Svobodová K., Cajthaml T. (2012). Laccase Activity Profiling and Gene Expression in PCB-Degrading Cultures of Trametes Versicolor. Int. Biodeterior. Biodegrad..

[15] Chun S.C., Muthu M., Hasan N., Tasneem S., Gopal J. (2019). Mycoremediation of PCBs by *Pleurotus ostreatus*: Possibilities and Prospects. Appl. Sci..

[16] Timothy M. (2017). PCB Dechlorination Hotspots and Reductive Dehalogenase Genes in Sediments from a Contaminated Wastewater Lagoon. Environ. Sci. Pollut. Res..

[17] Xiang Y., Xing Z., Liu J., Qin W., Huang X. (2020). Recent Advances in the Biodegradation of Polychlorinated Biphenyls. World J. Microbiol. Biotechnol..

[18] Adebusoye S.A., Picardal F.W., Ilori M.O., Amund O.O. (2008). Influence of Chlorobenzoic Acids on the Growth and Degradation Potentials of PCB-Degrading Microorganisms. World J. Microbiol. Biotechnol..

[19] Kumar A., Chandra R. (2020). Ligninolytic Enzymes and Its Mechanisms for Degradation of Lignocellulosic Waste in Environment. Heliyon.

[20] Hammel K.E., Tardone P.J. (1988). The Oxidative 4-Dechlorination of Polychlorinated Phenols Is Catalyzed by Extracellular Fungal Lignin Peroxidases. Biochemistry.

[21] Hirai H., Nakanishi S., Nishida T. (2004). Oxidative Dechlorination of Methoxychlor by Ligninolytic Enzymes from White-Rot Fungi. Chemosphere.

[22] Limura Y., Hartikainen P., Tatsumi K. (1996). Dechlorination of Tetrachloroguaiacol by Laccase of White-Rot Basidiomycete *Coriolus versicolor*. Appl. Microbiol. Biotechnol..

[23] Minard R.D., Liu S.-Y., Bollag J.M. (1981). Oligomers and Quinones from 2,4-Dichlorophenol. J. Agric. Food Chem..

[24] Roy-Arcand L., Archibaldt F.S. (1991). Direct Dechiorination of Chiorophenolic Compounds by Laccases from Trametes (Coriolus) Versicolor. Enzyme Microb. Technol..

[25] Valli K., Gold M.H. (1991). Degradation of 2,4-Dichlorophenol by the Lignin-Degrading Fungus *Phanerochaete chrysosporium*. J. Bacteriol..

[26] Li N., Xia Q., Niu M., Ping Q., Xiao H. (2018). Immobilizing Laccase on Different Species Wood Biochar to Remove the Chlorinated Biphenyl in Wastewater. Sci. Rep..

[27] Kordon K., Mikolasch A., Schauer F. (2010). Oxidative Dehalogenation of Chlorinated Hydroxybiphenyls by Laccases of White-Rot Fungi. Int. Biodeterior. Biodegrad..

[28] Nikolaivits E., Siaperas R., Agrafiotis A., Ouazzani J., Magoulas A., Gioti A., Topakas E. (2021). Functional and Transcriptomic Investigation of Laccase Activity in the Presence of PCB29 Identifies Two Novel Enzymes and the Multicopper Oxidase Repertoire of a Marine-Derived Fungus. Sci. Total Environ..

[29] Keum Y.S., Li Q.X. (2004). Fungal Laccase-Catalyzed Degradation of Hydroxy Polychlorinated Biphenyls. Chemosphere.

[30] Jiang G.X., Niu J.F., Zhang S.P., Zhang Z.Y., Xie B. (2008). Prediction of Biodegradation Rate Constants of Hydroxylated Polychlorinated Biphenyls by Fungal Laccases from *Trametes versicolor* and *Pleurotus ostreatus*. Bull. Environ. Contam. Toxicol..

[31] Germain J., Raveton M., Binet M.N., Mouhamadou B. (2021). Screening and Metabolic Potential of Fungal Strains Isolated from Contaminated Soil and Sediment in the Polychlorinated Biphenyl Degradation. Ecotoxicol. Environ. Saf..

[32] Sellami K., Couvert A., Nasrallah N., Maachi R., Abouseoud M., Amrane A. (2022). Peroxidase Enzymes as Green Catalysts for Bioremediation and Biotechnological Applications: A Review. Sci. Total Environ..

[33] Liu D., Coloe S., Baird R., Pedersen J. (2000). Rapid Mini-Preparation of Fungal DNA for PCR. J. Clin. Microbiol..

[34] Camacho C., Coulouris G., Avagyan V., Ma N., Papadopoulos J., Bealer K., Madden T.L. (2009). BLAST+: Architecture and Applications. BMC Bioinform..

[35] Abarenkov K., Tedersoo L., Nilsson R.H., Vellak K., Saar I., Veldre V., Parmasto E., Prous M., Aan A., Ots M. (2010). PlutoF—A Web Based Workbench for Ecological and Taxonomic Research, with an Online Implementation for Fungal ITS Sequences. Evol. Bioinform..

[36] Gouy M., Guindon S., Gascuel O. (2010). SeaView Version 4: A Multiplatform Graphical User Interface for Sequence Alignment and Phylogenetic Tree Building. Mol. Biol. Evol..

[37] Kellner H., Luis P., Schlitt B., Buscot F. (2009). Temporal Changes in Diversity and Expression Patterns of Fungal Laccase Genes within the Organic Horizon of a Brown Forest Soil. Soil Biol. Biochem..

[38] Kellner H., Luis P., Pecyna M.J., Barbi F., Kapturska D., Krüger D., Zak D.R., Marmeisse R., Vandenbol M., Hofrichter M. (2014). Widespread Occurrence of Expressed Fungal Secretory Peroxidases in Forest Soils. PLoS ONE.

[39] Barbi F., Bragalini C., Vallon L., Prudent E., Dubost A., Fraissinet-Tachet L., Marmeisse R., Luis P. (2014). PCR Primers to Study the Diversity of Expressed Fungal Genes Encoding Lignocellulolytic Enzymes in Soils Using High-Throughput Sequencing. PLoS ONE.

[40] Tian J.-H., Pourcher A.-M., Klingelschmitt F., Le Roux S., Peu P. (2016). Class P Dye-Decolorizing Peroxidase Gene: Degenerated Primers Design and Phylogenetic Analysis. J. Microbiol. Methods.

[41] Correa P.A., Lin L., Just C.L., Hu D., Hornbuckle K.C., Schnoor J.L., Van Aken B. (2010). The Effects of Individual PCB Congeners on the Soil Bacterial Community Structure and the Abundance of Biphenyl Dioxygenase Genes. Environ. Int..

[42] Galzy P., Slonimski P.P. (1957). Variations physiologiques de la levure au cours de la croissance sur l’acide lactique comme seule source de carbone. Comptes Rendus Hebd. Séances Académie Sci..

[43] Périgon S., Massier M., Germain J., Binet M.-N., Legay N., Mouhamadou B. (2019). Metabolic Adaptation of Fungal Strains in Response to Contamination by Polychlorinated Biphenyls. Environ. Sci. Pollut. Res..

[44] Mouhamadou B., Faure M., Sage L., Marçais J., Souard F., Geremia R.A. (2013). Potential of Autochthonous Fungal Strains Isolated from Contaminated Soils for Degradation of Polychlorinated Biphenyls. Fungal Biol..

[45] Fujihiro S., Higuchi R., Hisamatsu S., Sonoki S. (2009). Metabolism of Hydroxylated PCB Congeners by Cloned Laccase Isoforms. Appl. Microbiol. Biotechnol..

[46] Schultz A., Jonas U., Hammer E., Schauer F. (2001). Dehalogenation of Chlorinated Hydroxybiphenyls by Fungal Laccase. Appl. Environ. Microbiol..

[47] Gayosso-Canales M. (2012). PCBs Stimulate Laccase Production and Activity in *Pleurotus ostreatus* Thus Promoting Their Removal. Folia Microbiol..

[48] Sadañoski M.A., Benítez S.F., Fonseca M.I., Velázquez J.E., Zapata P.D., Levin L.N., Villalba L.L. (2019). Mycoremediation of High Concentrations of Polychlorinated Biphenyls with *Pleurotus sajor-caju* LBM 105 as an Effective and Cheap Treatment. J. Environ. Chem. Eng..

[49] Tandlich R., Bre B. (2001). The Effect of Terpenes on the Biodegradation of Polychlorinated Biphenyls by *Pseudomonas stutzeri*. Chemosphere.

[50] Villacieros M., Whelan C., Mackova M., Molgaard J., Sánchez-Contreras M., Lloret J., de Cárcer D.A., Oruezábal R.I., Bolaños L., Macek T. (2005). Polychlorinated Biphenyl Rhizoremediation by *Pseudomonas fluorescens* F113 Derivatives, Using a *Sinorhizobium meliloti* Nod System to Drive *Bph* Gene Expression. Appl. Environ. Microbiol..

[51] Kimura N., Watanabe T., Suenaga H., Fujihara H., Futagami T., Goto M., Hanada S., Hirose J. (2018). *Pseudomonas furukawaii* sp. Nov., a Polychlorinated Biphenyl-Degrading Bacterium Isolated from Biphenyl-Contaminated Soil in Japan. Int. J. Syst. Evol. Microbiol..

[52] Patel S., Gupta R.S. (2020). A Phylogenomic and Comparative Genomic Framework for Resolving the Polyphyly of the Genus *Bacillus*: Proposal for Six New Genera of *Bacillus* Species, *Peribacillus* Gen. Nov., *Cytobacillus* Gen. Nov., *Mesobacillus* Gen. Nov., *Neobacillus* Gen. Nov., *Metabacillus* Gen. Nov. and *Alkalihalobacillus* Gen. Nov. Int. J. Syst. Evol. Microbiol..

